# Analysis of bacterial communities of infected primary teeth in a Mexican population

**DOI:** 10.4317/medoral.23689

**Published:** 2020-08-27

**Authors:** Enid Karina Salas-López, Sergio Casas-Flores, Nguyen Esmeralda López-Lozano, Esther Layseca-Espinosa, Christian A. García-Sepúlveda, Perla del Carmen Niño-Moreno, Amaury Pozos-Guillén

**Affiliations:** 1 PhD, Facultad de Estomatología, Universidad Autónoma de San Luis Potosí, San Luis Potosí, México; 2 PhD, IPICYT, División de Biología Molecular, San Luis Potosí, México; 3 PhD, IPICYT, División de Ciencias Ambientales, San Luis Potosí, México; 4 PhD, Facultad de Medicina, Universidad Autónoma de San Luis Potosí, San Luis Potosí, México; 5 PhD, Facultad de Ciencias Químicas, Universidad Autónoma de San Luis Potosí, San Luis Potosí, México

## Abstract

**Background:**

The objective of this study was to describe the bacterial communities associated with pediatric patients with endodontic infections of temporal teeth by targeting the *16S * rRNA gene using pyrosequencing.

**Material and Methods:**

Microbiological samples were obtained from the lower primary molars of thirteen 13 pediatric patients with dental infections. An aspiration method for microbiological sampling was used. The identification of microbiota employing the pyrosequencing method by targeting the *16S * gene was performed.

**Results:**

Ribosomal *16S* RNA gene sequences were amplified, obtaining a total of 16,182 sequences from 13 primary infected molars (13 different individuals) by pyrosequencing. *Bacteroidetes* phyla (35.15%) were the most abundant followed by *Firmicutes* (33.3%) and *Fusobacteria* (10.05%); the presence of specific pathogenic bacteria was determined as well.

**Conclusions:**

The infected root canal of primary teeth contains a high diversity of anaerobic bacteria, and *Bacteroidetes*, *Firmicutes*, and *Fusobacteria* phyla were the most abundant; *Prevotella* and *Streptococcus* genera were the most prevalent.

** Key words:**Pyrosequencing, deciduous teeth, oral bacterial microbiota, 16S rRNA, taxonomy.

## Introduction

The invasion of microorganisms into the root canal system via dentinal tubules, mainly exposed by dental caries, represent a crucial role in endodontic/periapical infections; this situation is a common clinical problem in primary teeth. In severe cases, it may cause pain, swelling, abscesses, and even the premature loss of teeth. Microorganisms of the oral cavity have been described as oral microbiota, oral microflora, and recently, as the oral microbiome, which includes a community integrated by commensals, symbiotic, and pathogenic microorganisms, whose interactions can be related to the development of oral diseases such as caries and endodontic/periapical infections (1-3).

The relationship of microorganisms with endodontic pathologies has been recognized for more than 50 years, when pathologic changes were observed resulting from untreated experimental pulp exposures in germ-free rats as compared with conventional rats with a normally complex microflora (4). Until 1970, the most common bacterial group isolated by culture from the root canals of permanent teeth was *Viridans streptococcus*. Then, with the advance of strictly anaerobic culture techniques, the concept of endodontic infection changed due to that anaerobic microorganisms, which had been rarely isolated, were observed as the predominant endodontic microbiota in permanent teeth with necrotic pulp and periapical lesions (5). In general, a microbial variety and a spectrum of microbial communities related to endodontic infections have been described as primary, secondary, and persistent infections (6). Root canals could show different types of infections, and they are associated with different clinical conditions, which cause acute or chronic periradicular lesions. The composition of microbiota varies depending on the infection and the type of periradicular lesion. Overall, the infections are mixed, with the predominance of anaerobic and facultative bacteria (6,7).

In both primary and permanent teeth, several bacterial species, predominantly Gram-negative anaerobic microorganisms, have been similarly associated in the etiology of endodontic infections (8). However, the microbial profiles of permanent-tooth root canals have been evaluated to a greater extent, and these reports have shown a large variety of species, for example, *Lactobacillus*, *Veionella*, *Prevotella*, *Fusobacterium*, *Streptococcus*, *Tanerella*, *Bacteroidetes*, *Actinobacteria*, and others (6). Recently, through the development of anaerobic cultures and next-generation sequencing tools, it has been possible to identify aerobic and anaerobic microorganisms associated with infections in permanent teeth. However, few studies (9-13) have been addressed to identify the great diversity of microorganisms from primary teeth.

Pulp necrosis is commonly originated by caries, trauma, or other causes; in primary teeth, this condition might lead to periapical disease and could affect the permanent tooth germ. Pulp therapy in primary teeth with necrotic pulps aims at eliminating the infection and preventing early tooth loss (10). Bacteria play an important role on the establishment of pulp infection, and the main purpose of clinical treatment is the elimination of pulp infections from the root canal. It is indispensable to identify the pathogens isolated from infected primary teeth so that the appropriate antimicrobial agents can be utilized locally to eradicate these microorganisms. The objective of this study was to describe the bacterial communities associated with pediatric patients with endodontic infections of temporal teeth by targeting the <italic>16S</italic> rRNA gene using pyrosequencing.

## Material and Methods

- Patients

Patients were recruited from the Clinic for Pediatric Dentistry, Faculty of Dentistry, San Luis Potosi University, Mexico. The objective of the study was explained to either the parents or legal guardians, and written informed consent was obtained. This study included 13 patients (seven males and six females) between 4 and 7 years of age, who presented at the clinic for dental consultation and intervention. Inclusion criteria were children in good general health and with mandibular molars with carious lesion with or without direct exposure to the oral environment, containing at least one necrotic canal, abscess, or sinus tract. In addition, periapical x-rays of the selected teeth using the standard paralleling technique were taken to verify the presence of radiolucent area(s) in the furcation or periapical region and with at least two-thirds of the root remaining. Patients who had received antibiotics up to 4 weeks prior to sampling, who had used antimicrobial mouthwashes, and who presented with any systemic disease were excluded from the study. Demographic and clinical characteristics are shown in Table 1.

- Isolation, operative field disinfection, and microbiological sampling

After antisepsis of the oral cavity, local anesthesia was induced employing an infiltration. Each treated molar was cleaned and isolated with a rubber dam. Provisit (Casa Idea, SA de CV, México) was placed along the molar–rubber dam interface to prevent the leakage of saliva into the operative field. To disinfect the operative field, the following procedure was carried out in each tooth: the tooth crown, surrounding rubber dam, and clamp were swabbed with 30% H2O2 followed by 5.25% NaOCl for 1 min each. Caries was removed with a bur cooled with sterile saline solution; a sterile cotton pellet was placed on the floor of the chamber to prevent the penetration of disinfectants into the canals, and, with another sterile bur, the root canal was accessed.

**Table 1:**
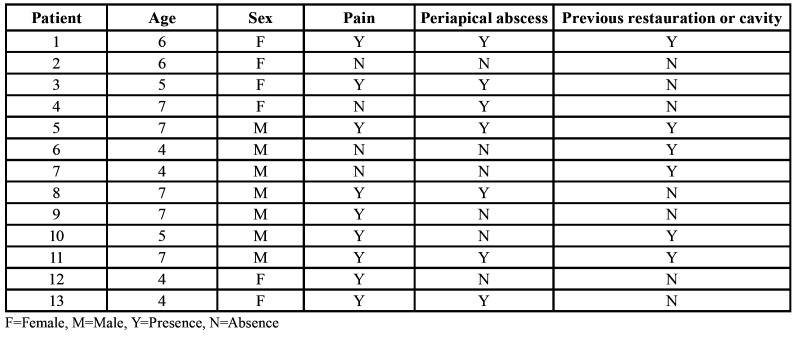
Demographic and clinical characteristics of the patients.

The cavity and field were again disinfected as described previously. The NaOCl was inactivated with 10% sodium thiosulfate for 1 min. Disinfection control samples were taken with sterile cotton pellets from the coronal surface of the tooth, rubber dam, and clamp, and immediately inoculated onto blood agar plates (BBL Becton Dickinson, México). The samples were then transferred to an aerobic incubator at 37ºC for 24-48 h (8). For sample collection, an endodontic file was passively introduced up to 1 mm from the radiographic apex or from the limit of root resorption and was gently moved in an apex-crown direction against the intracanal walls- After this, the file was removed and, using the washing and aspiration protocol with a plastic tip inserted into an electronic micropipette, the contents of the root canal was placed into a 1.5- mL polyethylene tube containing 1.0 mL of phosphate buffered saline (PBS) solution and snap-frozen at -80oC until analysis (14).

- Total DNA extraction of samples

Each sample was treated as described by Casas-Flores *et al*. (15); briefly, 200 μg of glass beads were added to each sample, and then 15 μL of lysozyme 20 mg/mL was added and mixed. Samples were incubated during 2h at 37ºC, and 5 μL of proteinase K and 50 μL of 20% SDS were added. Samples were incubated during 2 h at 65ºC and mixed at each 30-min interval. Thereafter, 1 volume of chloroform was added, centrifuged at 13,000 rpm for 10 min, and the aqueous phases were recovered, 1 volume of 70% isopropanol was added, and the mix was incubated overnight at -20ºC. Then, the samples were centrifuged during 20 min at 13,000 rpm, the supernatant was poured, and the pellet was washed with 750 μL 70% ethanol, centrifuged during 5 min at 13,000 rpm, and ethanol was eliminated by evaporation. The pellet was suspended in 20 μL of sterile Milli-Q H2O.

- PCR amplification and pyrosequencing

Each PCR reaction targeting the <italic>16S</italic> rRNA gene was performed using 0.3 μL each of <italic>16S</italic> rRNA universal primers 533F (16) and 1391R (17) at an initial concentration of 10 μM (Table 2).

**Table 2:**
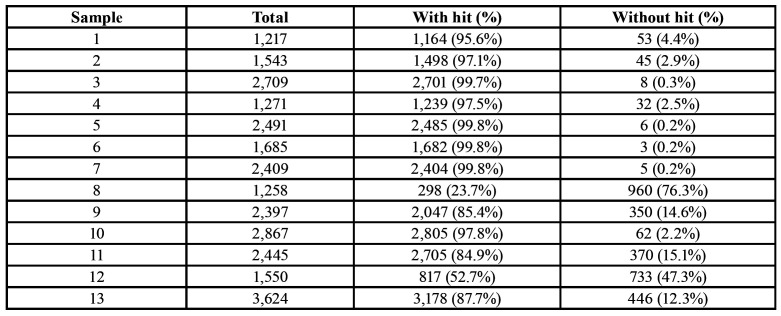
Total number of samples and total sequences processed.

Furthermore, 2.5 μL of 25 mM MgCl2, 0.125 U of Go Taq (Promega), 14.4 μL of sterile double-distilled water, and 0.4 of 10 mM dNTP were added. The reaction was performed in a GeneAmp system (Applied Biosystems) as follows: an initial denaturizing step at 94ºC during 2 min, followed by 30 cycles at 94ºCduring 1 min, 60ºC during 1 min, and 72ºC during 1:30 min, and completed with a final extension step at 72ºC during 7 min. Ten μL of each PCR product was resolved in a 1% agarose gel stained with ethidium bromide. A re-amplification of the PCR products was performed using the barcode primers of 454 Roche as recommended by the supplier. The re-amplified PCR products were purified by means of a MinElute PCR purification kit (Qiagen, Valencia, CA, USA), pooled, and sequenced in a 454 Life Sciences GS FLX system at the Laboratorio Nacional de Genómica para la Biodiversidad, CINVESTAV-Irapuato, Mexico, using the GS FLX Titanium Sequencing Kit XL, which allows for the sequencing of amplicons of 800 bp (base pairs).

- Data analysis

Mothur open source software (v.1.36.1) was utilized for the analysis of <italic>16S</italic> rRNA gene libraries. Sequences with homopolymer runs of eight or more bases, those with more than two mismatches with the sequencing primer, and a Q-value average below 25 were discarded. The potential occurrence of chimeric sequences was analyzed using the UCHIME algorithm. Sequences were aligned against the SILVA 123 <italic>16S</italic>/<italic>18S</italic> rRNA gene database, utilizing the Nearest alignment space termination (NAST) algorithm and trimmed for the optimal alignment region. With the non-redundant sequences, a pairwise distance matrix was calculated and reads were clustered into Operational taxonomic units (OTU) at a 3% distance using the furthest neighbor method. Mothur’s Bayesian classifier and the SILVA 123 reference set were employed to categorize taxonomically the sequences and the OTU. Statistical analysis, heat maps, and graphics were performed using the R statistical program (http://www.r-project.org/).

## Results

**Figure 1:**
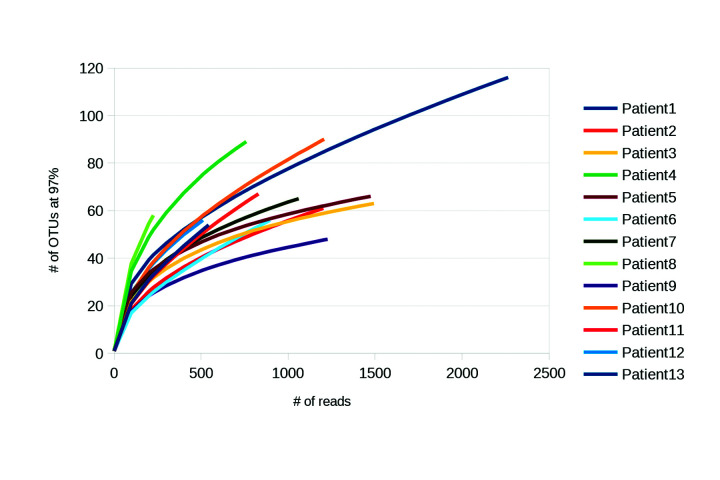
Rarefaction curves of bacterial OTU found in the infected primary teeth of Mexican patients. The readings were grouped into Operational taxonomic units (OTU) with a divergence of 3%.

A total of 16,182 sequences from 13 primary infected teeth were obtained by 454 pyrosequencing. These sequences were submitted to a quality control analysis to remove low-quality sequences and possible chimeras, obtaining 15,102 sequences, representing 93% of the total of sequences obtained with an average length of 800 bp. The number of reads by sample ranged from 583-2,240. Sequences that passed quality control were assigned to 889 OTU (Operational taxonomic units) by using a cutoff of 97% sequence identity (Table 2) (Fig. 1). The sample with the lowest number of OTU was from 54 (patient 1), whereas the highest number of OTU was from 116 for sample 13. In agreement with these results, the sample containing the highest number of reads demonstrated the highest number of OTU; however, that was not the rule, in that some samples with relative higher numbers of reads revealed low numbers of OTU (sample 3) and vice versa (sample 4). From the OTU, eight bacterial phyla were obtained with a relative abundance greater than 1%, and 23 bacterial genera with a relative abundance greater than 5% were assigned.

**Figure 2:**
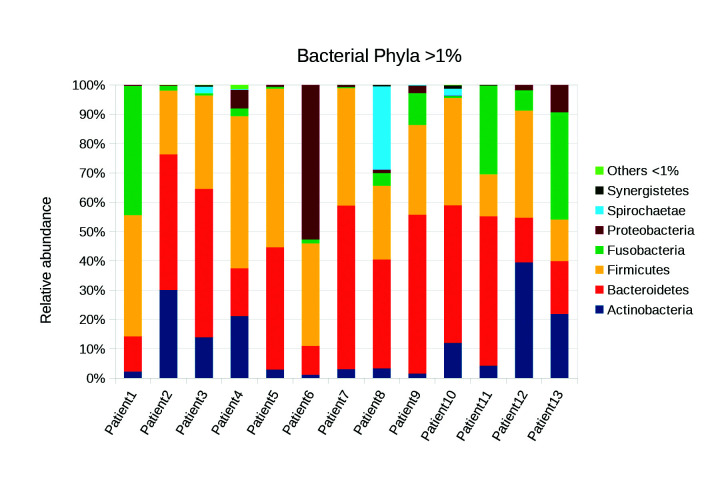
Bacterial phyla found in the infected primary teeth of 13 Mexican patients. The sample percentage of the relative abundance of each phylum of >1% in the libraries are shown.

**Figure 3:**
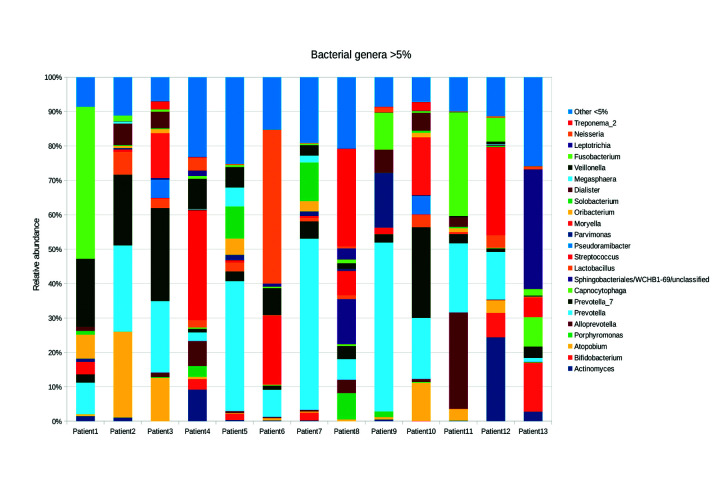
Bacterial genera found in the infected primary teeth of 13 Mexican patients. The percentages of the relative abundance of each genus of >5% are shown.

Eight phyla were found in the 13 teeth (Fig. 2). Regarding relative abundance, the *Bacteroidetes* phyla (35.15%) was the most abundant, followed by *Firmicutes* (33.3%) and *Fusobacteria* (10.05%) and, found at lesser amounts were *Actinobateria* (13.5%), *Spirochetes* (2.6%), and *Synergist* (5.3%). A total of 2,035 genera were found; the most abundant genera at a relatively high abundance were *Prevotella* and *Streptococcus* (Fig. 3).

## Discussion

The oral cavity has one of the highest amounts of microorganisms, mainly bacteria, distributed among more than 700 species. Bacteria are the main etiologic agents of primary endodontic infections. In these infections, multiple species have frequently been detected, predominantly anaerobic bacteria. Over 500 different bacterial species have been identified in infected root canals by different methods, such as culture, conventional PCR molecular tools, and new-generation sequencing, including pyrosequencing (18,19). There are numerous methods for evaluating the root canal microbiome; however, they possess low or limited coverage. Pyrosequencing is a deep-coverage sequencing technique; the 454 pyrosequencing technology facilitates metagenomic study through a high-throughput, deep-coverage, massively parallel, and multiplex barcoded approach (20).

Anaerobic bacteria are those most commonly isolated from endodontic infections, mainly Gram-positive anaerobic cocci, such as *Peptostreptococcus anaerobius*, *Peptostreptococcus micros*, and Gram-negative anaerobic rods, including *Porphyromonas endodontalis*, *Porphyromonas *gingivalis**, *Prevotella intermedia*/nigrescens, *Prevotella melaninogenica*, *Fusobacterium nucleatum*, and *Fusobacterium necrophorum *(21).

In this study, up to 88.8% of the total of different bacteria identified in the different samples exhibited a higher amount of species compared with those recently obtained by pyrosequencing. For instance, Li *et al*. (22) found 43 species in a total of nine teeth, whereas Ribeiro *et al*. (23) described 70 species. Another study with nine teeth reported five phyla and contained 17 genera and 105 species (24). Santos *et al*. (25) used <italic>16S</italic> rRNA, to identify endodontic bacteria and detected *Firmicutes* as the most abundant phylum, followed by *Bacteroidetes*. In the present study, *Bacteroidetes* was the most abundant phylum with 29.6%, followed by *Firmicutes* with 23.2%, and *Fusobacteria* with 13.1%.

To our knowledge, there are few studies describing the microbiota in primary teeth (5,8-13). In this regard, it was reported that 10 bacterial species are commonly found in temporal teeth, *Fusobacterium nucleotum* and *Prevotella intermedia* the most abundant species in endodontic infections (13). In other recent study, Yun *et al*. (11) analyzed 10 infected deciduous teeth by means of pyrosequencing, yielding a total of 64,291 <italic>16S</italic> rRNA gene sequences; the most abundant phylum was Proteobacteria (50.4%), followed by *Firmicutes* and *Bacteroidetes* with 25.8 and 9.5%, respectively, and 187 genera, determining that *Neisseria*, *Streptococcus*, and *Veionella* were the most abundant genera. Regarding genera, the results of these authors are in agreement with those of the present study, because the authors found *Streptococcus* and *Veionella* at a higher amount. However, the authors found lower percentages of the *Prevotella* genus, which is in disagreement with the present results. *Fusobacterium* is associated with severe endodontic infection and with polymicrobial infection (26). Also, is recognized the ability of *Fusobacterium* to intensify the pyogenic capacity of *Streptococcus constellatus* strains and to suppress their phagocytic killing by human polymorphonuclear leukocytes (27). On the other hand, bacterial combinations of *Porphyromonas*, *Parvimonas* micra, and *Fusobacterium* are related with pain intensity (28).

In studies of this nature, bacterial recovery is essential, for which different methods have been used, and the bacterial recovery capacity may depend on the sampling method. In order to reduce the weaknesses of sampling by using paper points, this study used a novel method involving the washing/aspirating of the root-canal (14). This method reduces the exposure of the microorganisms to the external environment. It is suggested that with this technique, the microbial load is greater and allows a greater recovery of microorganisms (number and variety). In addition, using a negative control on all of the samples ensures that the microorganisms found were exclusively from the root canal and not transferred by the rubber dam.

More studies with temporal teeth are needed in order to have a comparative parameter, because the microorganisms reported are strictly aerobic, and more and more microorganisms resistant to antibiotics are being identified every day; uncontrolled use of antibiotics, and self-medication of antibiotics aggravates this clinical problem (29). In this respect, the pharmacological treatment for these patients must be considered, as well as the cleaning of the conduct of the tooth at the moment of the pulp therapy.

Also, more studies are needed to evaluate variables such as the ethnic origin, feeding habits, hygiene, oral health, type of infection, sociodemographic context, among others. It is important to know the inhabiting microorganisms of infected temporal teeth, which could be giving rise to and/or aggravating a persistent infection in a different part of the human body. Additionally, given the great variety of endodontic microorganisms and their diverse virulence factors, the exact microbial species can be determined in terms of whether any specific group of bacteria is associated with specific endodontic symptoms and clinical signs (30).

Finally, the results revealed the presence of combinations of bacterial species, with a majority of anaerobic bacteria, in the root canal of infected primary teeth. It is necessary to carry out specific studies that include hygiene habits, diet, sex, and different living conditions in order to establish an association among microorganisms. These observations suggest investigating their possible clinical implications and the designing of novel therapeutic strategies in the field of irrigating solutions and filling materials employed in endodontic treatments as disinfectants.

## Conclusions

The infected root canal of primary teeth contains a high diversity of anaerobic bacteria, and *Bacteroidetes*, *Firmicutes*, and *Fusobacteria* phyla were the most abundant of these. The genera *Prevotella* and *Streptococcus* were the most prevalent.
